# When Down Is Up: Heterochromatin, Nuclear Organization and X Upregulation

**DOI:** 10.3390/cells10123416

**Published:** 2021-12-04

**Authors:** Reem Makki, Victoria H. Meller

**Affiliations:** Department of Biological Sciences, Wayne State University, Detroit, MI 48202, USA; reem.makki@wayne.edu

**Keywords:** heterochromatin, dosage compensation

## Abstract

Organisms with highly differentiated sex chromosomes face an imbalance in X-linked gene dosage. Male *Drosophila* solve this problem by increasing expression from virtually every gene on their single X chromosome, a process known as dosage compensation. This involves a ribonucleoprotein complex that is recruited to active, X-linked genes to remodel chromatin and increase expression. Interestingly, the male X chromosome is also enriched for several proteins associated with heterochromatin. Furthermore, the polytenized male X is selectively disrupted by the loss of factors involved in repression, silencing, heterochromatin formation or chromatin remodeling. Mutations in many of these factors preferentially reduce male survival or enhance the lethality of mutations that prevent normal recognition of the X chromosome. The involvement of primarily repressive factors in a process that elevates expression has long been puzzling. Interestingly, recent work suggests that the siRNA pathway, often associated with heterochromatin formation and repression, also helps the dosage compensation machinery identify the X chromosome. In light of this finding, we revisit the evidence that links nuclear organization and heterochromatin to regulation of the male X chromosome.

## 1. Maintaining Appropriate Ratios of Gene Dosage Is Vital for Cells and Organisms

Cells require precise levels of proteins, and this is particularly important for multi-subunit complexes. Even small deviations of one subunit from normal levels may degrade complex function, cause aggregation of unassembled proteins and produce cellular stress [1]. Although gain or loss of a single copy of one gene is usually without phenotype, the cumulative effect when many genes are unbalanced by chromosomal aneuploidy can be fatal. For this reason, highly differentiated sex chromosomes pose a challenge to the survival of one sex. In flies and humans, females have two gene-rich X chromosomes and males have one X and a gene-poor Y chromosome. Although the mechanisms used to balance gene expression are very different in flies and mammals, each selectively modulates expression from a single chromosome to maintain a consistent ratio of X to autosomal gene products in both sexes [2,3].

In eutherian mammals, dosage compensation is achieved by inactivating one of the two X chromosomes in female cells during early embryonic development [4]. X-inactivation is controlled by a locus on the X chromosome called the X-inactivation center (XIC). The XIC contains the X-inactive specific transcript (Xist) gene, which encodes a long non-coding RNA. The Xist transcript is responsible for triggering silencing *in cis* [5]. This is accompanied by sequential eviction of RNA polymerase, recruitment of repressive factors and deacetylation of histones on the inactivated X chromosome, ultimately establishing a durable inactive state [6,7]. This process is accompanied by a chromosome-wide structural reorganization and relocation to the nuclear periphery [8,9].

The roundworm *Caenorhabditis elegans* also compensates by repression, but in this organism, hermaphrodites (XX) repress transcription from both X chromosomes [10]. This is accomplished by a condensin-like Dosage Compensation Complex (DCC) that is recruited to both X chromosomes [11]. This complex contains proteins specific to dosage compensation as well as proteins that function in mitotic and meiotic chromosome segregation [12].

**Figure 1:**
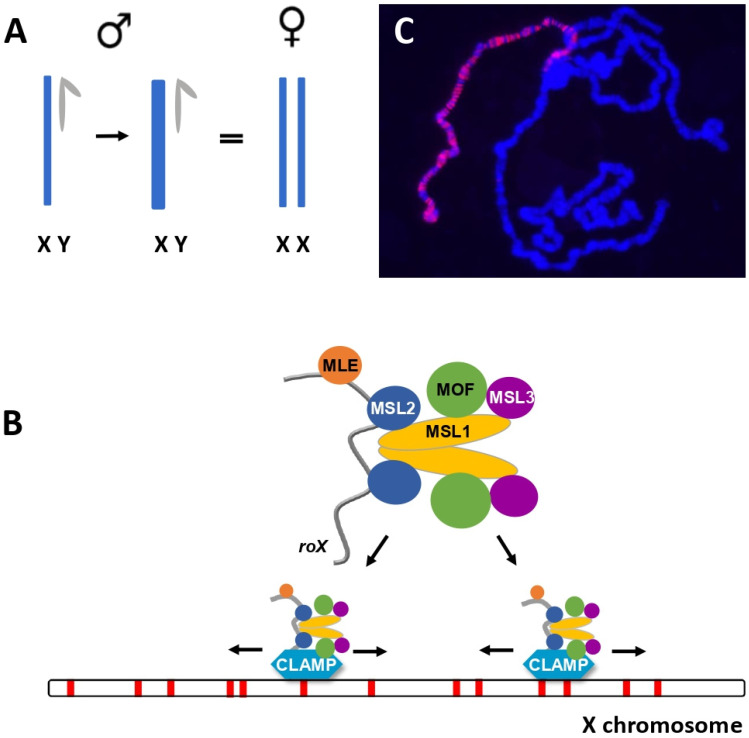
Dosage compensation in *Drosophila* equalizes sex chromosome expression between males and females. (**A**) *Drosophila* males have one X chromosome while females have two. Males increase transcription from their X-linked genes approximately two-fold to equalize expression. (**B**) The Male Specific Lethal (MSL) complex, composed of five proteins and a long non-coding RNA, is recruited to CLAMP-bound Chromatin Entry Sites (red) on the X chromosome. The MSL complex then spreads *in cis* to nearby transcribed genes by recognition of active chromatin marks. (**C**) Polytene chromosome preparation from a wild type male. MSL2, detected by Texas Red, identifies the X chromosome. DNA is counterstained with DAPI (blue).

In contrast to mammals and *C. elegans*, *Drosophila* males increase expression from their X chromosome two-fold in somatic cells (Figure 1A). The well-studied Male Specific Lethal complex (MSL complex, also known as the Dosage Compensation Complex or DCC), is essential for this process (Figure 1B,C). Composed of five proteins and a long, non-coding *roX* RNA, the MSL complex is recruited to active genes on the X chromosome where it acetylates histone 4 on lysine 16 (H4K16ac) [13,14]. Enrichment for H4K16ac decondenses chromatin and elevates levels of gene expression by facilitating elongation [15,16,17,18]. Low levels of enrichment around the promoter may also increase initiation [19]. Replacement of H4 by H4K16R blocks H4K16ac and has a strikingly male-biased lethality, demonstrating the essential role of this modification in a male-limited process [20].

## 2. Subunits of the MSL Complex Determine Localization and Chromatin Modification

The core of the MSL complex is formed by the Male Specific Lethal 1 and 2 proteins (MSL1, MSL2). Loss of either MSL1 or MSL2 eliminates X chromosome binding by the remaining complex proteins [21]. MSL2 is the only strictly male-limited component of the MSL complex [22,23,24]. Expression of MSL2 during early development triggers assembly of intact complexes that localize to the male X chromosome [25]. MSL1 dimerizes and serves as a scaffold with binding sites for MSL2, Males absent on the First (MOF), and Male Specific Lethal 3 (MSL3) [26,27,28,29]. Mutations that block MSL1 dimerization or interact with other MSL proteins inactivate the MSL complex. MSL3 contains a chromodomain that binds the co-transcriptional H3K36me3 mark and is necessary for enrichment of the MSL complex within the body of active genes [17,30]. MOF is the histone acetyltransferase responsible for enrichment of H4K16ac on the male X chromosome [31,32,33]. The fifth MSL protein, Maleless (MLE), is an RNA/DNA helicase that binds the non-coding *roX* RNAs and is presumed to associate with the other proteins through an RNA tether [34,35].

The X-linked *roX1* and *roX2* genes produce long non-coding RNAs (lncRNAs) that are dissimilar in size and sequence but functionally redundant for dosage compensation [36]. Loss of either *roX* transcript alone is without obvious phenotype, but loss of both is male lethal. In moribund *roX1 roX2* male larvae the MSL proteins continue to associate but relocalize to ectopic autosomal sites, most notably, heterochromatic regions such as the chromocenter and the 4th chromosome [37,38]. *roX1* and *roX2* have little similarity but small inverted repeats near the 3′ end of each transcript share homology and are conserved in closely related species [39]. These form stem loops that are essential for *roX* function [40]. Remodeling of the stem loops by MLE creates an alternative base pairing that enables MSL2 binding and integrates *roX* into the MSL complex [41,42,43]. MSL3 and MOF are reported to bind RNA but, unlike MLE and MSL2, show no great preference for *roX* transcripts [44,45,46]. Nonspecific RNA-binding by other members of the complex may promote recruitment to regions of active transcription or contribute to phase separation of the dosage compensated X chromosome [47]. While it is clear that *roX* transcripts are critical for exclusive localization of the X chromosome, exactly how *roX* achieves this remains unknown.

## 3. Compensated X Chromosomes Establish Distinct Nuclear Compartments

The remarkable selectivity of the MSL complex for the X chromosome has been the subject of considerable investigation and speculation. The combined action of recruiting elements, local spreading and phase separation convert the dosage compensated male X chromosome into a unique subnuclear domain with distinctive organization, location and epigenetic marks [47,48,49,50]. The principles that guide X recognition and compensation are not limited to a single species as compensated X chromosomes of mammals and *Caenorhabditis elegans* also form subnuclear domains with organization and localization that are distinct from the autosomes [12,51].

The mammalian X chromosome often associates with the nuclear envelope [52] or with the nucleolus periphery [53]. Association of the inactive X (Xi) with the Lamin B receptor facilitates spreading of Xist across the X chromosome and silencing of transcription [9]. Association with the perinucleolar periphery is also dependent on Xist and maintains the Xi in its repressive chromatin state [53]. In addition, the Xi exhibits a distinct three-dimensional structure compared to the active X (Xa), which is also dependent on Xist RNA [54]. The Xi shows a smoother and rounder shape, a higher compaction of some segments and the absence of long-range interactions between silenced loci, suggesting that the chromatin and nuclear territory of the inactivated X are subject to a dramatic reorganization compared to other chromosomes [54,55,56]. These features, and the observation that Xist interacts with several proteins known or predicted to participate in phase separated bodies, support the idea that phase separation drives the inactivation and unique structure of the Xi [57].

Similar to the mammalian system, compensation in *C. elegans* is accompanied by a change in chromatin architecture and a relocation of the compensated chromosomes to a distinct region of the nucleus [58,59]. Compared to autosomes that interact strongly with the nuclear lamina, both hermaphrodite X chromosomes are only loosely attached to the periphery [60,61]. In contrast, the single male X is more frequently located at the nuclear periphery than other chromosomes [58]). The organization and localization of the hermaphrodite X chromosomes is in part regulated by the DCC, which binds to recruitment sites followed by spreading along the chromosomes. This results in weakening of peripheral localization and more compacted chromosomal territories. DCC mutations alter the topology of the compensated X chromosomes to a conformation resembling that of autosomes [59,62,63,64].

While mammalian females silence one of their two X chromosomes and *C. elegans* hermaphrodites reduce expression of both X chromosomes, male flies elevate expression of their single X chromosome. Despite this distinction, repressive factors do have roles in dosage compensation in flies; current evidence suggests that these are likely to contribute to X recognition or the characteristic organization of the compensated male X.

## 4. How Does the MSL Complex Find the X?

A few hundred specialized sites on the X chromosome retain partial MSL complexes in *msl3 or mle* mutant backgrounds [65,66]. These have been termed Chromatin Entry Sites (CES) or High Affinity Sites (HAS). Analysis of sequences that retain MSL2 in a *msl3* mutant background identified GA-rich MSL Recognition Sequences, or MREs, that are enriched approximately 2-fold on the X chromosome and necessary for recruitment by the CES [67,68]. A subgroup of CES, termed PionX, with extended binding sites enable an earlier recruitment of the complex [69]. A protein that binds MREs, Chromatin-linked adapter protein (CLAMP), was subsequently identified in a search for factors necessary for X localization of MSL proteins [70]. Surprisingly, CLAMP is essential in both males and females, and so must have vital functions outside of dosage compensation [71]. CLAMP binds MREs throughout the genome in both sexes but only recruits the MSL complex to specialized CES on the X chromosome. Insertion of a CES on an autosome enables recruitment of MSL complex and upregulation of nearby genes, revealing that the CES fulfills a function in addition to CLAMP binding [67,72,73,74]. The answer may lie in MSL2, which has a DNA-binding CXC domain that is necessary for X chromosome association and interacts with the minor groove of the MRE [27,29,75]. Simultaneous binding of CLAMP and MSL2 is thought to distinguish CES [76].

Interactions between CES establish a higher order architecture unique to the male X chromosome that facilitates long-range spreading [48,77]. After recruitment to CES, the MSL complex spreads to active genes nearby [73,78,79]. Local spreading relies on the MSL3 chromodomain, which binds the co-transcriptional H3K36me3 mark [30,80]. MSL complex enrichment is most prominent in the body of highly transcribed genes and closely follows the profile of H3K36me3 accumulation [81,82]. One consequence is that strongly expressed genes are more perfectly compensated in suboptimal situations, for example, when autosomal genes are in proximity to a transgene containing recruiting elements [83].

## 5. The *roX* Genes Have Multiple, Intertwined Roles in X Recognition

Not only are the *roX* genes the source of a subunit of MSL complex, but both *roX* genes are X-linked and overlap CES [67,68]. As initial assembly of the MSL complex is proposed to occur on nascent *roX* transcripts, proximity to a CES would facilitate local chromatin association [39,73,78]. The proximity of assembly to entry sites suggested that the *roX* genes could mark the X chromosome. This idea was particularly appealing as the mammalian lncRNA Xist, which originates from the inactive female X chromosome and initiates silencing, functions strictly *in cis* [84]. Autosomal *roX* transgenes recruit the MSL complex to their insertion site and induce local spreading of the MSL complex into nearby genes [73]. But, unlike Xist, *roX* RNA is not limited to action *in cis*. *roX* RNA transcribed from an autosomal transgene supports X localization of the MSL proteins and rescues *roX1 roX2* males to adulthood [36]. Furthermore, the redundancy provided by hundreds of CES distributed across the X chromosome ensures that loss of the *roX*-associated CES is without obvious phenotype. While the capacity of *roX* genes to recruit dosage compensation *in cis* is striking, the situation of *roX* genes on the X chromosome optimizes function but is not essential.

## 6. Satellite Repeats, Epigenetic Modifications and X Recognition

Additional features distinguish the X chromosome and contribute to localization of the MSL complex. Hundreds of copies of an AT-rich repetitive sequence, the 1.688^X^ satellite repeats, are strikingly enriched on the X chromosome. These are also termed the 1.688 g/cm^3^ repeats (CsCl density) or 359 bp repeats (unit length) [85,86]. Unlike most repetitive DNA, the 1.688^X^ repeats are dispersed in short tandem clusters in euchromatin [87]. They are often situated close to or within genes on the X chromosome, leading to the proposition that they play a role in modulation of expression [88]. In addition, the X chromosome has large blocks of related repeats near the telomere and comprising approximately 10 Mb of pericentric heterochromatin [89]. When a few 1.688^X^ repeat units are integrated on an autosome, the MSL complex is recruited to nearby actively transcribed genes and expression is increased in males [83]. 1.688^X^ chromatin does not generally attract high levels of MSL complex binding and these repeats are dissimilar in sequence to the CES, suggesting that they function in a different manner.

Many 1.688^X^ repeats are transcribed and siRNA originating from these repeats has been detected in flies, suggesting the potential for small RNA-directed chromatin modification [90,91]. The extreme redundancy of 1.688^X^ sequences on the X chromosome makes probing their function by deletion impossible. To determine if 1.688^X^ siRNA influences X recognition, flies expressing double-stranded hairpin 1.688^X^ RNA were generated and found to contain high levels of siRNA [91]. Remarkably, ectopic 1.688^X^ siRNA partially restored X localization of MSL2 and enabled recovery of 30% of adult males with lethal *roX1 roX2* chromosomes. Single stranded 1.688^X^ RNA had no effect, or enhanced the male lethality of partial loss of function *roX1 roX2* chromosomes. This suggests that a siRNA-dependent system contributes to the function of the X-linked 1.688^X^ satellite repeats. Supporting this idea, recruitment of compensation *in cis* to autosomal 1.688^X^ insertions was enhanced by 1.688^X^ siRNA [83]. However, recruitment by a *roX1* transgene was not, revealing a key genetic difference in how 1.688^X^ repeats and CES-containing *roX* genes recruit dosage compensation.

In accord with a role of small RNA in X recognition, mutations in several siRNA pathway members act to enhance *roX1 roX2* male lethality (Table 1). Reduction in proteins necessary for siRNA production, including Dicer 1 and -2, enhance *roX1 roX2* lethality [92]. Reduction of the effector protein Argonaute 2 (Ago2) further reduces MSL recruitment to the X chromosome in *roX1 roX2* males. Reduction of Ago2, or of several Ago2-interacting proteins including the H3K9 methyltransferase *Su(var)3-9*, also reduce the survival of *roX1 roX2* males [93]. *Su(var)3-9* is responsible for enrichment of H3K9me2 at many 1.688^X^ repeats and ectopic 1.688^X^ siRNA increases H3K9me2 enrichment around autosomal insertions of 1.688^X^ DNA. In spite of the well-known repressive role of H3K9me2, enrichment over autosomal 1.688^X^ transgenes is associated with increased expression of genes up to 140 kb away in male larvae [93]. Taken together, these studies suggest that siRNA-directed chromatin modification at 1.688^X^ repeats contributes to X recognition. How a repressive chromatin mark participates in a process that elevates expression from active genes remains unclear.

## 7. Proteins That Bind RNA Interact Genetically with *roX1 roX2*

**Figure 2:**
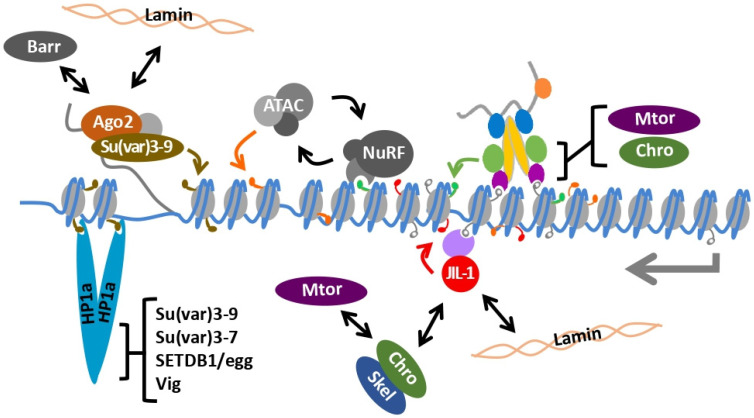
Genes that participate in heterochromatin formation modulate dosage compensation and X chromosome structure. (**Left**) Genes involved in heterochromatin establishment or the siRNA pathway. Ago2 recruits Su(var)3-9 to nascent transcripts in an siRNA-dependent manner, leading to enrichment of H3K9me2/3 (brown modification) and HP1a binding. Ago2 interacts with Barr, nuclear lamins and many other proteins, including HP1a. HP1a interacts with several other proteins that enhance *roX1 roX2* male lethality. (**Right**) Proteins that interact with the dosage compensation machinery. Transcribed regions (gray arrow) are enriched for H3K36me3 (gray modification). Both the MSL complex (**Top**) and JASPER/JIL-1 (**Bottom**) bind H3K36me3 and are enriched in active genes. MOF acetylates H4K16 (green modifications). MOF and MSL3 interact with Chro, Mtor and several additional nuclear pore proteins (not shown). JIL-1 phosphorylates H3S10 (red modification), a mark enriched on the male X chromosome, and interacts with lamins, Chro and Skeletor. (**Middle**) H4K16ac enhances chromatin binding of the ISWI-containing NURF complex. The Gcn5-containing ATAC complex Interacts with NURF, acetylates chromatin (orange modifications) and may also modify NURF. Interactions are validated but may not be direct.

Genetic screens for enhancement of *roX1 roX2* lethality identified several additional genes with roles in small RNA silencing and chromatin organization (Table 1) [92,93]. These include the RNA-binding protein *vasa intronic gene* (*vig*), which interacts with HP1a, Ago1 and Ago2 [101,102]. Mutations in *fmr1*, an RNA-binding protein and possible RITS complex component, and *smg*, encoding an RNA-binding protein that modulates translation and interacts with Ago1 and Ago2, also enhance *roX1 roX2* male lethality [123]. The condensin subunit *barren* (*barr*) is necessary for proper chromosome segregation and interacts with small RNA factors, including Ago2 and spn-E [104]. Mutation of *barr* similarly enhances *roX1 roX2* male lethality. A recurring theme shared by these factors is the ability to bind RNA or interact with small RNA effector proteins. Some possible intersections between these factors and the dosage compensation machinery are depicted in Figure 2. But, as many of these proteins have extensive interaction networks, the basis of phenotypic enhancement may be complex. It is important to note that limited, directed screens of candidates identified the genes presented in Table 1. It is expected that many additional factors capable of enhancing *roX1 roX2* male lethality have yet to be identified.

## 8. Heterochromatin and the Male X Chromosome

Discovery of a link between repressive marks and the dosage compensated male X chromosome was unexpected but far from unusual. In fact, the structure of the polytenized male X chromosome is selectively disrupted by under- or overexpression of any one of several proteins with established roles in heterochromatin formation (Table 1). Reduced levels of HP1a disorganize banding of the male polytene X chromosome, producing a short, bloated chromosome [107]. HP1a is also modestly enriched along the male X [124,125,126]. The morphology of the polytene male X chromosome is similarly affected by reduction of Su(var)3-9 and by reduction or overexpression of Su(var)3-7, a heterochromatin protein that interacts with HP1a and Su(var)3-9 [107,108,127]. The bloated X phenotype of Su(var)3-7 or Su(var)3-9 mutants is rescued by mutations in MSL complex members, revealing a genetic interaction with dosage compensation [107]. Reduced levels of Su(var)3-7 cause displacement of the MSL complex to the chromocenter and acetylation of H4K16 in these regions [109]. Conversely, when Su(var)3-7 is overexpressed, the MSL proteins and H4K16ac become mislocalized to autosomal regions. Although extreme overexpression disrupts banding of all chromosomes, the male X is most sensitive.

In wild type males the MSL complex is exclusive to X euchromatin, but manipulation of the levels of components of this complex produce ectopic localization, often to heterochromatic regions. For example, overexpression of MSL1 and MSL2 produces ectopic binding of MSL proteins at autosomal sites and the chromocenter [128]. Furthermore, males with *roX1 roX2* mutations have reduced localization of the MSL proteins to the X chromosome but accumulation at autosomal sites, most prominently the 4th chromosome and chromocenter [36,38]. This is accompanied by a shift in H4K16ac from the X chromosome to the fourth chromosome and chromocenter [37]. The basis of the affinity of MSL proteins for heterochromatin remains unresolved.

## 9. Chromatin Remodeling and the Male X Chromosome

The male X chromosome is similarly sensitive to partial loss of function mutations in the NURF and ATAC complexes, which act to restore regular nucleosome arrays and acetylate histones, respectively [113,129]. Loss of NURF subunits *Nurf301* or the *ISWI* ATPase disrupt the architecture of the male X, which appears less condensed than normal, something not seen in females [110,111,130]. Binding of the MSL complex and acetylation of the male X chromosome is retained in both mutants. Normal polytenized structure is restored by mutation of *mof* or both *roX* genes, demonstrating that disruption of polytene structure requires the activity of the MSL complex [110,111]. Mutations in the Gcn5 histone acetyltransferase or the Ada2a subunit of the ATAC complex also selectively disrupt the polytenized male X chromosome [114]. Once more, the X chromosome phenotype is dependent on the action of the MSL complex. The NURF and ATAC complexes regulate common targets and cooperative interactions between these complexes have been noted (Figure 2; [114]). Disruption of the male X by loss of ATAC or NURF function may thus involve the same molecular pathway.

## 10. The Dual kinase JIL-1 Maintains Interphase Chromatin Structure

The male X is enriched for H3Ser10 phosphorylation (H3S10p) catalyzed by the dual kinase JIL-1 [118]. JIL-1 forms a heterodimer with Jasper, a protein that binds H3K36me3, the co-transcriptional mark also recognized by MSL3 [131]. This results in an enrichment of JIL-1 in the bodies of transcribed genes in a pattern overlapping that of the MSL complex. Pull down of chromatin-bound MSL complex followed by mass spec identified JIL-1, a finding explained by the proximity of these factors [132]. Loss of JIL-1 preferentially reduces male survival and, similar to the factors already described, induces shortening of polytenized chromosome arms and disruption of banding [118,119]. More severe JIL-1 alleles disrupt all chromosomes, but the dosage compensated male X is most sensitive. JIL-1 also interacts with proteins enriched in interbands, Chromotor (Chro) and Skeletor [133,134]. In accord with a role in nuclear organization, JIL-1 is reported to interact with nuclear lamins [135]. Association with active chromatin may be the basis of another role of JIL-1, enforcement of barriers between heterochromatin and euchromatin [136]. In JIL-1 mutants, pericentric H3K9me2 spreads into euchromatic chromosome arms, most prominently on the X chromosome. Interestingly, spreading of H3K9me2 into the X chromosome is also observed in females and must therefore be independent of the activity of the MSL complex. In addition to their interphase roles, JIL-1, Chro and Skeletor dissociate from chromosomes during mitosis and, along with another JIL-1 interacting protein Megator (Mtor), assemble with the spindle matrix [137]. It is possible that some interactions noted in Figure 2 only occur in mitotic cells.

## 11. A Governor to Limit over Activation?

Many of the factors described above have been linked to repression. It is tempting to speculate that they act to limit overexpression. However, the genes that have been found to limit activation by the MSL complex lack an obvious link to heterochromatin. One is *over compensating males* (*ocm*), a protein with a role in blood cell differentiation that interacts with transcription factors and Polycomb Group proteins [115,138,139]. The other is *Megator* (*mtor*), which, in addition to association with JIL-1, has been identified in pull downs of the MSL complex [50]. Mtor is a component of the nuclear pore basket but is not limited to the nuclear periphery, associating with active chromatin during interphase and the spindle matrix during mitosis [140,141]. In accord with roles in limiting activation, mutations of *ocm* or *mtor* suppress the male lethality of *msl* or *roX1 roX2* mutants [115,117]. In contrast, reductions in *Su(var)3-9*, *Su(var)3-7*, or siRNA pathway genes enhance *roX1 roX2* male lethality [92,93]. This reveals that, rather than limiting activation, these factors support normal compensation of the male X chromosome.

## 12. Heterochromatin and Dosage Compensation Are Integrated on the 4th Chromosome

A role for repressive factors in the context of dosage compensation is not unique to the X chromosome. The small 4th chromosome is a fine-grained mosaic of heterochromatin interspersed with euchromatin, while other fly chromosomes have a more defined segregation of heterochromatin to telomeres and pericentric regions [142]. In spite of the autosomal status of the 4th chromosome, it is capable of dosage compensation and adults that are hemizygous for the fourth are recovered. The discovery that the 4th chromosome is an ancestral X chromosome suggests that an ancient dosage compensation system retains function [143]. Interestingly, the Painting of Fourth (POF) protein, which coats the 4th chromosome in a manner reminiscent of the MSL proteins on the X chromosome, is necessary for survival of flies with a single 4th chromosome [144,145]. POF binds nascent RNA and is enriched within gene bodies [146]. Surprisingly, POF also interacts with HP1a and the SETDB1/egg H3K9 methyltransferase, both strikingly enriched on the 4th chromosome [147,148]. Although methylation on H3K9 is associated with repression, genome-wide examination of SETDB1/egg localization suggests that, unlike Su(var)3-9, it tends to associate with active genes and insulators that are frequently unmethylated [149].

The HP1a and POF binding profiles overlap and HP1a requires POF for wild type levels of recruitment to the 4th chromosome [144]. Remarkably, depletion of either POF or HP1a reduces expression of 4th-linked genes [146,150]. Both studies identify a characteristic, low pausing index for 4th linked genes that is disrupted by loss of HP1a or POF, suggesting that enrichment of these proteins within gene bodies enables the transcriptional machinery to advance. Facilitation of transcript elongation is a strategy similar to that employed by the MSL complex. While H4K16ac enrichment by the MSL complex is associated with chromatin de-condensation and elevated expression, how HP1a and POF modulate transcription is unknown. HP1a has also been shown to bind RNA during induced transcription of highly expressed genes and exert a positive effect on expression in this context [151]. Despite the enrichment of HP1a on the male X chromosome and the role of this protein in maintaining organization of the polytenized male X, a genetic interaction with *roX1 roX2* has not yet been detected, and the role of this protein in dosage compensation remains speculative.

## 13. Genetic Interactions between *roX* Genes and Male Heterochromatin

Loss or reduction in heterochromatin proteins often reduces the expression of genes that reside in and rely on a heterochromatic environment [152]. Interestingly, genes in autosomal heterochromatin decrease in expression in *roX1 roX2* males [153]. Reduced expression is particularly pronounced on the 4th chromosome, where nearly every gene is reduced by up to 50%. Loss of *roX* RNA does not affect localization of POF or the survival of flies with a single 4th chromosome, eliminating the possibility of a dual role for *roX* RNA in dosage compensation of the X and 4th chromosomes. Providing further evidence of heterochromatin disruption is a striking suppression of position effect variegation (PEV) in *roX1 roX2* males. Surprisingly, this is not due to mislocalization of MSL proteins. Mislocalization of these proteins and H4K16ac enrichment in heterochromatic regions is recapitulated in *roX1 roX2* females forced to express MSL2, but no misregulation of heterochromatic genes or suppression of PEV occurs in these females [153]. Suppression of PEV in *roX1 roX2* males does not require a Y chromosome or the absence of Sexlethal (SXL), the master regulator of sexual differentiation. Instead, it appears to be regulated by the number of X chromosomes. Specifically, deletion of pericentric 359 repeats from the female X chromosome, or mutation of topoisomerase 2, a protein enriched at these repeats, “masculinizes” female heterochromatin and enables suppression of PEV in *roX1 roX2* females [154]. These studies support the idea that heterochromatin is sexually dimorphic, and that components of the dosage compensation system are necessary for normal heterochromatin function in males. The precise mechanism is unclear, but as maternally provided MSL proteins bind throughout the genome in early embryos, it is possible that they participate in establishment of sexually dimorphic heterochromatin [155]. These studies highlight the affinity of MSL proteins for heterochromatin and the complex, multifaceted relationship between dosage compensation and heterochromatin.

## 14. Full Compensation Involves Multiple Mechanisms

The MSL complex does not act alone. Inactivation of the MSL complex results in only partial loss of dosage compensation. X-linked gene expression in S2 cells is reduced by 22–40% following knock down of MSL2 and expression in male larvae mutated for both *roX* RNAs is reduced by 26% [37,79,82]. Additional mechanisms must therefore contribute to full compensation. A leading candidate is a genome-wide system that buffers aneuploidy, sometimes referred to as autosomal compensation [2,156]. Our understanding of autosomal compensation is based on descriptive studies that have identified features of genes, or rearrangements, that make them more or less subject to buffering in response to changes in copy number [157,158]. Despite the absence of molecular detail about this process, features of aneuploidy buffering suggest that systems of repression could play a role. Genes with low expression that are situated in repressive regions associated with the nuclear envelope are most effectively compensated in response to loss of a homolog [159]. These authors propose that unpaired regions are released from the nuclear envelope and relocate to regions more favorable for transcription. Buffering genes with low expression could complement the effect of the MSL complex. Recruitment and acetylation by the MSL complex is robust for X-linked genes that exceed a minimum expression level [14]; however, autosomal genes near integrated recruiting elements are only efficiently compensated when highly expressed [83].

Other evidence points to a stage-specific mechanism for compensation. Zygotic transcripts from some X-linked genes are compensated in embryos prior to the formation of the MSL complex [160]. Many X-linked genes are unaffected, but dose-sensitive developmental genes that participate in embryo patterning are modulated. A mechanistically different post transcriptional mechanism is suggested by the observation that mRNAs of many X-linked genes, some with developmental roles, have SXL binding sites in their untranslated regions [22]. This is intriguing as SXL binding to the MSL1 and MSL2 messages achieves partial or full repression of translation in females. Taken together, these findings support the idea that full compensation is achieved by the combined action of the MSL complex in males, a genome-wide but poorly understood buffering system, translational repression in females and adjustment of transcript levels from X-linked developmental genes in early embryos. It is likely that individual genes are compensated by different mechanisms depending on developmental stage, sex and tissue.

## 15. Systems of Dosage Compensation Converge on Nuclear Organization

Dosage compensation occurs within a highly structured nucleus and intersects with virtually every aspect of chromatin regulation. Mutations that influence histone modification, nucleosome remodeling, heterochromatin formation and small RNA selectively disrupt the male X chromosome or interact genetically with mutations that degrade MSL complex function. Other genes that influence compensation highlight a potential role for elements of higher order nuclear organization, including insulators, boundary elements, the lamina and nuclear pore proteins. Disruption of the structure of the polytenized male X chromosome upon reduction of many of these proteins is dependent on MSL complex activity. This striking concurrence suggests that these factors influence a common pathway, but the diversity of function of the many factors sharing this phenotype confounds a simple hypothesis.

The repressive nature of many genes associated with disruption of the polytenized male X chromosome suggests a system to limit upregulation. In contrast to this idea, reduction in the function of several heterochromatin proteins, or the siRNA pathway, enhances the male lethality of *roX1 roX2* mutations. This leads us to the counterintuitive conclusion that these genes promote, rather than limit, dosage compensation of the X chromosome. We propose that the involvement of several repressive factors in *Drosophila* dosage compensation stems, in part, from the involvement of the siRNA pathway and 1.688^X^ repeats in X chromosome identification [91]. This does not rule out additional roles for heterochromatic factors in maintaining normal structure of the X chromosome and fails to explain the well-documented affinity for MSL proteins and heterochromatin. Interestingly, genes that have been found to limit X upregulation have no apparent association with heterochromatin. One of these, *ocm*, interacts with Polycomb Group proteins and transcription factors with developmental roles. The other, *mtor*, has multiple roles as a nuclear pore protein, a component of active interphase chromatin and as part of the mitotic spindle matrix. There is currently no clear link between Ocm and Mtor, and the mechanism through which they limit X activation is unknown.

Although the strategy of sex chromosome compensation is very different in other organisms, the important role of nuclear organization in modulation of an entire chromosome is a common feature of this process. For example, inactivation of a mammalian X chromosome takes advantage of preexisting long-range contacts and the X chromosome territory [51]. Nuclear pore proteins, and the position of compensated X chromosomes in the nucleus, contributes to modulation of expression in *C. elegans* [58]. Understanding of how these systems are integrated to achieve the robust and selective modulation of an entire chromosome is an important future goal.

## Figures and Tables

**Table 1:** Genes that preferentially disrupt the male X chromosome or interact genetically with *roX1 roX2*. Genes are grouped by macromolecular complex or molecular process. Many of these are primarily associated with heterochromatin. Mutations that enhance the *roX1 roX2* phenotype are often in genes necessary for siRNA production or in the RITS effector complex.

Complex or Process	Gene	Functions	Mutant Phenotype	Citations
Small RNA production or action	Ago2	siRNA slicer nuclease	Enhances *roX1 roX2* male lethality	[92,93,94]
	Rm62	RNA helicase, RNA processing		[93,95]
	Dcr1	Small RNA processing		[92,93,96,97]
	Dcr2	Small RNA processing		[92,93,97,98]
	Fmr1	RNA-binding, translational regulation		[93,95]
	Elp1	RNAPII elongation, binds Ago2 Dcr-1,-2		[92,99]
	Loqs	dsRNA-binding, siRNA processing		[92,100]
	vig	Interacts with Ago1, Ago2 and HP1a		[93,101,102,103]
	barr	Interacts with Ago2 and spn-E		[93,104]
	Smg	RNA-binding, translation, mRNA stability, miRNA production		[93,105,106]
Heterochromatin	Su(var)3-9	H3K9 methyltransferaseHeterochromatin formation	Polytenized male X disorganized Enhances *roX1 roX2* male lethality	[93,107]
	Su(var)3-7	Heterochromatin formation		[93,107,108,109]
	HP1a	H3K9me2/3 binding Heterochromatin formation	Polytenized male X disorganized	[107]
NURF complex	ISWI	ATP-dependent nucleosome remodeler	Polytenized male X disorganized Enhances *roX1 roX2* male lethality	[110,111,112], Meller lab unpublished
	Nurf301	Nucleosome remodeling	Polytenized male X disorganized	[111]
ATAC complex	Gcn5	Histone acetyltransferase	Polytenized male X disorganized	[113,114]
	Ada2a	Chromatin binding		[113,114]
Limit compensation	Ocm	Polycomb group interactions	Suppresses *roX1 roX2* male lethality	[115,116]
	Mtor	Nuclear pore subunit		[117]
Misc.	JIL-1	Dual kinase, boundary elementEnriched on male X chromosome	Polytenized male X disorganized	[118,119]
	upSET	Maintains heterochromatin Binds MSL3, HDAC1 and SIN3-A	Enhances *roX1 roX2* male lethality	[93,120,121,122]
